# Neutrophil recruitment and function mediate sexual dimorphism in experimental periodontitis

**DOI:** 10.3389/fdmed.2026.1974918

**Published:** 2026-10-14

**Authors:** Kelsey Martin, Victoria Maglaras, Maxwell J. Mianecki, Asfandyar Sheikh, Ridhi Gupta, Rana S. Dabaja, Muhammad H. A. Saleh, Ann M. Decker, Joseph T. Decker

**Affiliations:** 1 Department of Periodontics and Oral Medicine, University of Michigan School of Dentistry, Ann Arbor, MI, United States; 2 Department of Cariology, Restorative Sciences, and Endodontics, University of Michigan School of Dentistry, Ann Arbor, MI, United States

**Keywords:** alveolar bone, inflammation, neutrophils, periodontitis, sex differences

## Abstract

Periodontitis is a chronic inflammatory disease that has been shown to have biological sex-based differences with respect to disease prevalence and severity. Understanding the mechanisms behind this difference may highlight potential adjunctive therapies for periodontal disease that could address differences in disease progression between males and females. Our objective was to examine clinical and immunological differences between male and female mice in periodontal disease progression using the ligature-induced periodontitis model. Periodontitis was induced in male and female C57BL/6J mice for 7 or 21 days, at which point maxillae were characterized for bone loss by microCT or immune infiltrate by flow cytometry. Ligature-induced periodontitis led to alveolar bone loss at both 7 and 21 days in both female and male mice. Males and females had similar linear bone loss 7 days post-ligature placement, while male mice had significantly more linear bone loss by day 21. Male mice had significantly more immune cells in their maxillae 7 days post-ligature placement compared to female mice (*p* < 0.05). Both male and female mice showed a shift in immune populations towards neutrophils, with no significant difference between males and females. Neutrophil counts were elevated in male mice on day 7 but not day 21, while female mice did not have any statistically significant changes in neutrophil counts. Neutrophil depletion using a Ly6G antibody limited bone loss in male but not female mice relative to isotype antibody-treated controls. Analysis of single-cell sequencing data from human patients with periodontitis showed that, similar to males in our mouse model, male patients with periodontitis showed enrichment for CXCR4 expression with low CD62L (SELL). Padi4 knockout in male and female mice had decreased bone loss at 21 days post-ligature compared with WTs and still displayed sexual dimorphism. Together, these data suggest a mechanistic role for neutrophil inflammation in sexual dimorphism in periodontitis.

## Introduction

Periodontal disease is a debilitating inflammatory condition within the supporting tissues of the teeth (including gingiva, alveolar bone, and the periodontal ligament) that leads to significant morbidity, systemic consequences, and economic difficulties for patients (1–7). Periodontal disease is extremely common; a recent NHANES stratified multistage probability sample of the civilian noninstitutionalized population assessment reported that 42% of the US population has some type of periodontitis (severe, moderate, or mild), including 7.8% being severe periodontitis as defined by the CDC/AAP periodontitis case definitions (8). Furthermore, the disease burden of periodontal disease has significant consequences for overall health, as periodontal disease has been associated with numerous comorbidities, including diabetes, cardiovascular disease, adverse pregnancy outcomes, rheumatoid arthritis, and chronic obstructive pulmonary disease (4–6). Understanding the patient-specific mechanistic underpinnings of periodontal disease is essential for both oral and systemic health.

Biological sex is one such patient-specific mechanism that may contribute to the overall prevalence and severity of periodontal disease. Periodontal disease is more common in men compared to women (8–13). Additional studies mechanistically attribute this sex-biased presentation to behavior patterns, habits, and attitudes, which form a part of environmental risk factors rather than constitutional factors (14, 15). However, there is newer evidence that this previously held belief is incorrect and that there exists a biological foundation to the sexually dimorphic epidemiological observation (16–19), where social determinants can only explain some of the observed sexual dimorphism in the prevalence and severity of periodontal disease (20).

Sexual dimorphism has been widely observed in the clinical prevalence of periodontitis, yet very few mechanistic studies have been performed to elucidate the underlying mechanisms for these differences. Therefore, a need exists to dissect the specific biology underpinning sexual dimorphism in periodontitis. The primary goal of this study was, therefore, to define the contribution of biological sex to the progression of periodontal disease in a preclinical model system and identify potential immunological mechanisms underpinning sexual dimorphism in this disease.

## Methods & materials

### Animals

This study used male and female C57BL/6J mice or Padi4 knockout mice on a C57BL/6J background (Jackson Laboratories, Bar Harbor, Maine). Mice were maintained according to institutional animal care and use guidelines, and experimental protocols were approved by the Institutional Animal Care and Use Committee of the University of Michigan (PRO00010967 and PRO00011223). Mice were housed in groups of 2-5 under specific pathogen-free conditions and randomly assigned to control or experimental groups. Mice were excluded if they experienced significant trauma during ligature placement, were found to be missing a ligature at the time of euthanasia, or lost a tooth to periodontal disease. All experiments were conducted with *n* = 5-8 per group, with conditions predominantly separated by cage.

### Ligature-induced periodontitis

Periodontitis was induced in mice using the previously described ligature-induced periodontitis model (21). Mice six to eight weeks old were anesthetized with a ketamine/xylazine cocktail following isoflurane vaporizer induction, and a 5-0 silk suture (Roboz, Gaithersburg, MD) was tied around the maxillary left second molar. The ligature was left on for 7 or 21 days before being euthanized to evaluate periodontitis progression.

### Micro-computed tomography

Mice were euthanized through CO_2_ asphyxiation followed by cervical dislocation. Maxillae were harvested, fixed in 10% buffered formalin for 24-72 hours at 4°C on a rocker, and stored in phosphate-buffered saline. Specimens were scanned using a micro-CT system (µCT100 Scanco Medical, Bassersdorf, Switzerland). Scan settings for maxillae were: 34 mm sample holder, voxel size 18 µm, 70 kVp, 114 µA, 0.5 mm AL filter, and integration time 500 ms. Images were analyzed by a single calibrated, blinded examiner using Dragonfly (version 2024.1, Comet Technologies). Distance from the cemento-enamel junction to the alveolar ridge was measured at four sites (mesial, buccal, distal, and palatal) at the midline of the second molar (22). Bone loss was calculated as the difference between average bone levels between ligated and unligated sides of the same mouse. Volumetric measurements were calculated as the volume from 10 slices below the cemento-enamel junction (approximate height of healthy bone) to the alveolar bone crest (22).

### Histology

Maxillae were fixed in 10% buffered formalin for 24-72 hours at 4°C on a rocker and stored in 70% ethanol. Samples were placed into labeled tissue cassettes and decalcified in a 14% EDTA solution for 2 weeks. All cassettes were processed quickly using a VIP tissue processor. Samples were paraffin-embedded using a Leica paraffin embedding machine, and sagittal sections were cut at 5 µm. Sections were stained with hematoxylin and eosin for bone and cellular identification.

### Trap staining and quantification

Histological sections were stained using TRAP to identify osteoclasts per the manufacturer's protocol (Casom Bio Inc, Tokyo, Japan). Osteoclasts were counted manually using the entirety of a 4x image centered on the ligated tooth. Positive staining was included in the quantification if it was highly concentrated and contained multiple nuclei. Two calibrated individuals performed the counting, and data were reported as the average of the two counts.

### Immunostaining

Slides were deparaffinized, followed by antigen retrieval using Proteinase K (Ambion catalog no. AM2548). Sections were stained with primary antibodies for Ly6G [Rat mAb to Ly6g + Ly6c (RB6-8C5), Abcam, United Kingdom] and/or citrullinated Histone H3 (E403F, Cell Signaling, MA, United States) overnight. Samples were mounted with ProLong Gold with DAPI (Invitrogen, CA, United States).

### Flow cytometry

Maxillae were harvested and kept in RPMI media until all samples were collected. Maxillae were cut in half through the palate, at the end of the hard palate, and before the incisor. Maxillae blocks were transferred into digestion media containing DNase1 (0.1 mg/mL; ThermoScientific J62229.MC) and Liberase (0.1 mg/mL; Sigma-Aldrich 0540154001) and incubated at 37°C for 30 minutes, minced, incubated for another 30 minutes, and digestion was stopped with 2700 µL complete media. Tissue and media were filtered using a 70 µm cell strainer, centrifuged for 5 minutes at 4°C, the supernatant was decanted, and cells were resuspended before being transferred to microcentrifuge tubes. Peripheral blood (to assess neutrophil depletion) was lysed using 2.5 mL of ACK lysis buffer (Life Technologies Corporation) and incubated for 10 minutes before centrifugation and washing. Samples were incubated for 10 minutes with flow cytometry antibodies. The following antibodies and dilutions were used: PE/Cy7- CD49b 1:250, AlexaFluor 700- F4/80, PerCP/Cy5.5- CD4 1:250, PerCP- CD44 1:250, BV711- CXCR4 1:250, BV510- CD19 1:250, BV605- NK1.1 1:250, FITC- CD11b 1:250, BV785-CD11c 1:250, PE- CD45R/B220 1:250, BV570- CD62L, BV421- Ly6G 1:250; Biolegend; BV480- CD8a 1:250, NovaFluor Yellow 730- CD3a 1:50, Invitrogen; APC- Ly6C 1:250; BD Bioscience, and live/dead stain (Fixability Dye eFluor450 1:1000; eBioscience). Samples were analyzed using a Cytek Northern Lights (3 laser; V-B-R). Data were unmixed using single-color controls in SpectroFlow software. Data was processed using FlowJo v 10.8.1 (Becton Dickinson). The gating scheme used to identify cell types can be seen in Supplementary Figure S1.

### Neutrophil depletion

200 µL anti-Ly6G (Bio X Cell, clone 1A8, catalog no. BE0075-1) or Rat 1gG2a isotype control (Bio X Cell, catalog no. BE0089) (0.168 mg/mL) was injected intraperitoneally every other day for 22 days, beginning on Day −1. Retro-orbital blood was collected on Day 4 to confirm neutrophil depletion via flow cytometry. Immunostaining confirming depletion of neutrophils in the maxilla can be seen in Supplementary Figure S2.

### Single-cell sequencing analysis

Publicly available human single-cell RNA sequencing datasets – GSE164241 (23) and GSE171213 (24)– of patients with (*n* = 7 male, 6 female) and without (*n* = 5 male, 12 female) periodontitis were collected for analysis. Patient files included for evaluation were those collected from gingival tissues of adults (≥ 18 years of age) in good general health and who had not taken anti-inflammatory or antibacterial drugs within 3 months. Inclusion criteria were similar between the two studies, with patients in the moderate-to-severe periodontitis groups having probing depths over 5 mm and bleeding on periodontal probing. Analyses were performed in R, utilizing the Seurat v3 package (25). Dataset files were first downloaded from GEO Accession Viewer. GSE164241 files were input using ‘Read10X’ and GSE 171213 files were input utilizing ‘read_table’ followed by ‘read.delim’ to create a Seurat-compatible format using the ‘CreateSeuratObject’ function. Files were merged using the ‘merge’ function into groups based on biological sex and periodontal condition – healthy female, healthy male, periodontitis female, and periodontitis male – for subsequent evaluation. Quality control, normalization, and integration were performed as previously described (23, 25). Visualization and clustering were accomplished using 'ScaleData' to account for the cell cycle, followed by ‘FindNeighbors’ and ‘FindClusters’. Markers for each cluster, identifying unique cell types, were identified using ‘FindMarkers’, and in totality using ‘FindAllMarkers’ (only.pos = TRUE, logfc.threshold = 0.25, min.pct = 0.5), based on differential gene expression. Cell types were identified through a literature search for highly upregulated marker genes or through gene ontology. UMAP and heatmap plots were produced to reflect highly expressed markers of interest by cluster. Relative expression plots of cell-specific subtypes were generated to discern expression differences between the four groups. Cell-cell communication networks were inferred using CellChat (26). Gene ontology was performed using MetaScape (27).

### Statistical analysis

Statistical analysis was performed using GraphPad Prism version 10.3.0 for Windows (GraphPad Software, Boston, MA). Bone loss measurements and flow cytometry results were compared using two-way ANOVA followed by Tukey's multiple comparison correction unless otherwise noted. Single comparisons were analyzed by *t*-test. A p-value < 0.05 was used for statistical significance in all studies. All experiments were performed on *n* ≥ 5 mice.

## Results

### Male and female mice have different amounts of bone loss in chronic periodontitis

We began our study of sexual dimorphism in experimental periodontitis by comparing the progression of ligature-induced periodontitis (LIP) in male and female C57BL/6J mice (Figure 1). Male and female mice had their teeth ligated, and alveolar bone levels at the site of the ligature were compared at days 7 and 21 post-ligature placement (Figure 1A). These time points were selected to mimic the progression of periodontal disease in humans (28), with day 7 approximating acute inflammation and day 21 approximating chronic inflammation. Male and female mice had similar levels of alveolar bone prior to ligature placement as measured by µCT (Figures 1B,C). Ligature-induced periodontitis led to alveolar bone loss at both 7 and 21 days in both female and male mice (Figures 1D,E). Males and females had approximately the same amount of linear bone loss at 7 days post-ligature placement (0.20 mm male, 0.22 mm female, *p* = 0.79, Figure 1D). Male mice had significantly more linear bone loss in the 21-day model of chronic periodontitis (0.60 mm male, 0.46 mm female, *p* < 0.0001, Figure 1D). Measuring the volume of lost bone showed similar results, with similar bone loss 7 days post ligature placement (0.33 mm^3^ male, 0.33 mm^3^ female, *p* > 0.9999) and greater bone loss in male mice 21 days post ligature placement (0.90 mm^3^ male, 0.67 mm^3^ female, *p* < 0.001, Figure 1E). TRAP staining of maxillae during periodontitis (Figure 1F) showed similar numbers of TRA*P* + cells at day 7, with elevated TRAP + cells in female mice by day 21 (Figure 1G).

**Figure 1:**
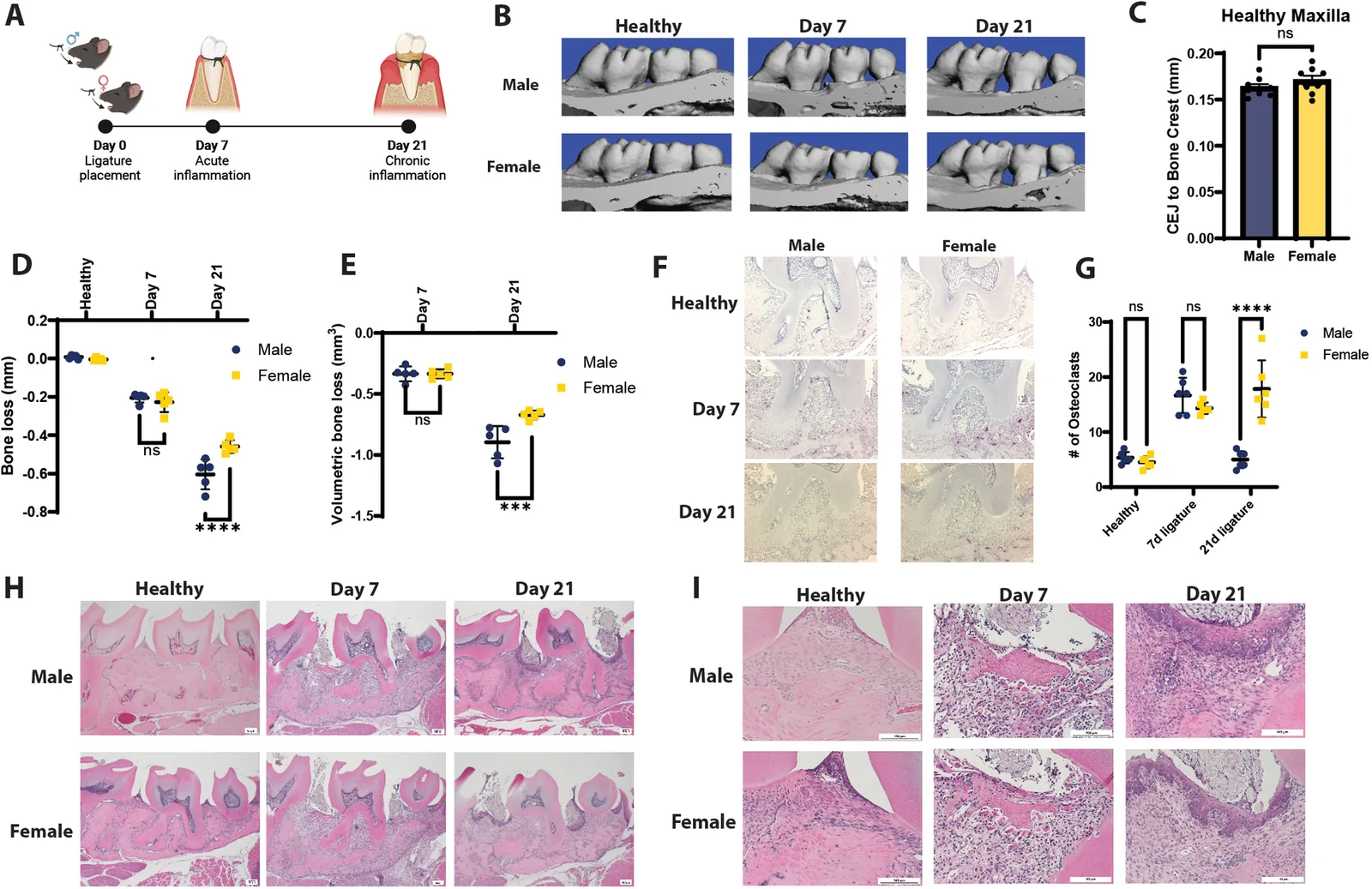
Sexual dimorphism in the clinical phenotype of experimental periodontitis. **(A)** Time course of ligature-induced periodontitis. **(B)** MicroCT images of healthy, day 7, and day 21 maxillae in male and female mice. **(C)** Cemento-enamel junction (CEJ) to alveolar bone crest distances in healthy male and female mice. **(D)** Linear bone loss measurements in male and female mice at the healthy, day 7, and day 21 time points. **(E)** Volumetric bone loss in male and female mice at the day 7 and day 21 time points. **(F)** TRAP staining images from healthy, day 7, and day d1 maxillae of male and female mice. **(G)** Quantification of TRAP + cells in the maxillae of male and female mice. **(H)** Histology images of the entire ligature site in male and female mice. Scale bar = 100 µm. **(I)** High-magnification image of mucosal surfaces between the first and second molars. Scale bar = 50 µm. ns = not significant. ***p < 0.001, ****p < 0.0001, two-way ANOVA with Tukey multiple comparison correction. Data are presented with bars indicating the mean value ± standard deviation.

In healthy male and female mice, the gingival sulcus around the teeth is shallow (Figures 1H,I). Histologically, the gingival sulcus is lined by the sulcular epithelium, attached at the most apical aspect to the enamel surface. The alveolar bone is well-defined with distinct and intact borders. At the day 7 time point, histological staining depicts that the junctional epithelium has detached from the tooth surface, forming an intra-epithelial cleft with increased permeability. The pocket epithelium shows heavy infiltration, particularly of the epithelial ridges, with neutrophilic granulocytes and lymphocytes. The alveolar bone is not clearly linear on the coronal and lateral borders. At the day 21 time point, proliferation of the epithelial ridges into the soft connective tissue is noted, with very thin regions between these ridges. Focal micro-ulcerations of the epithelial ridges are noted at the free surface of the pocket epithelium. Additional interdental alveolar bone loss has occurred beyond 50% of the root surface length.

### Neutrophil infiltrate is exaggerated in male mice with periodontitis

The inflammatory response is a primary driver of bone loss in periodontitis. We therefore characterized the changes in the immune cell populations during LIP in male and female mice, with the hypothesis that male and female mice would have differential recruitment of immune cells (Figure 2). Male and female mice had similar numbers of immune cells in the maxilla prior to ligature placement (Figure 2A). Male mice had significantly more immune cells in their maxilla 7 days post-ligature placement relative to female mice (*p* < 0.05); this difference in total immune cell count was resolved by day 21. Both male and female mice showed a shift in immune populations towards Cd11b^+^ Ly6G^+^ neutrophils, with no significant difference between males and females (Figure 2B). Counts of immune cell populations showed significantly more CD11b^+^ Ly6G^+^ neutrophils (*p* < 0.01) and CD19^+^ B cells (*p* < 0.05) in male maxillae 7 days post ligature placement (Figure 2C). The total number of neutrophils, but not B cells, was elevated in male maxillae 21 days post ligature placement compared with female mice (*p* < 0.0001, Figure 2D). We further analyzed changes in neutrophil infiltrate over time (Figures 2E,F,*G*). Male mice showed a significant trend toward increased neutrophil fraction of immune cells (fraction of CD45 + cells) over time, while female mice showed a similar, though not statistically significant, trend (Figure 2F). Both male and female mice showed a peak in neutrophil counts in the maxilla at day 7, with only male mice having a statistically significant change from healthy (*p* < 0.001). Immunostaining for Ly6G^+^ neutrophils in the maxillae of male and female mice showed similar results (Figures 2H,I).

**Figure 2:**
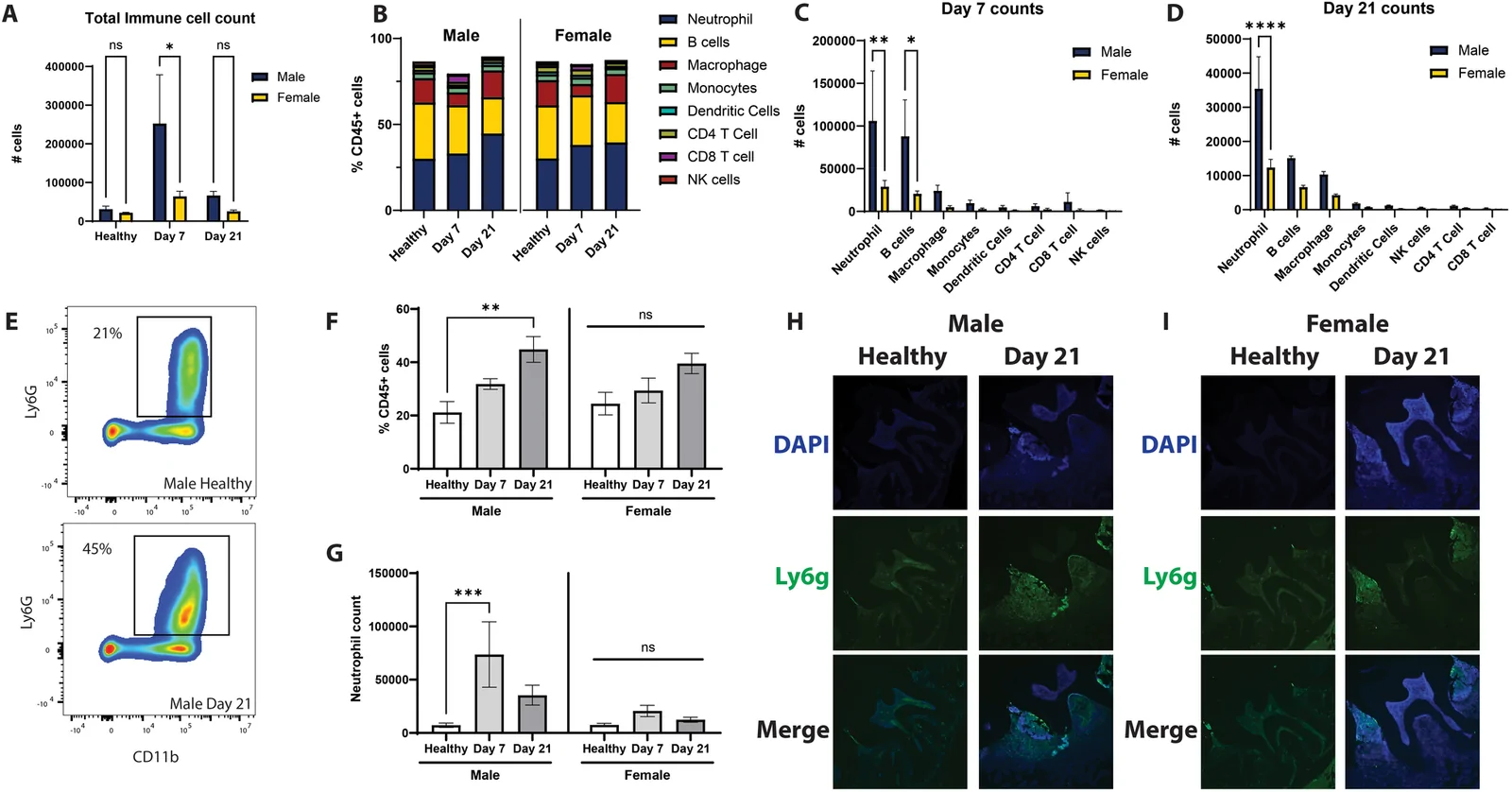
Increased neutrophil infiltrate in male mice with periodontitis. **(A)** Total immune cell counts in the maxilla in male and female mice at different time points of LIP. **(B)** Proportions of CD45^+^ cells by immune cell type in the maxilla. **(C)** Counts of different immune cell populations in the maxilla at day 7. **(D)** Counts of different immune cell populations in the maxilla at day 21. **(E)** Example flow plot showing increased neutrophil infiltrate in the day 21 male maxilla. **(F)** Trends towards increasing neutrophil fraction over time in the maxilla of male but not female mice. **(G)** Peak neutrophil infiltrate at day 7. **(H)** Immunostaining images of Ly6G^+^ cells in the maxillae of male mice. **(I)** Immunostaining images of Ly6G^+^ cells in the maxillae of female mice. NS = not significant. * *p* < 0.05, **p < 0.01, ***p < 0.001, ****p < 0.0001, two-way ANOVA with Tukey's multiple comparison correction.

### Neutrophil depletion differentially affects male and female mice with periodontitis

Since neutrophil inflammation persists in male mice relative to female mice, we next examined the sexual dimorphism in bone loss during periodontitis in mice depleted of neutrophils (Figure 3A). Systemic treatment with a Ly6G antibody successfully depleted neutrophils in the blood of both male and female mice relative to isotype antibody-treated controls (Figures 3B,C). Mice treated with an isotype antibody showed similar amounts of bone loss relative to untreated controls, with significantly less bone loss in female mice compared with male mice at the day 21 time point (0.51 mm male, 0.44 mm female, *p* < 0.05, Figures 3D,E). Sexual dimorphism was also observed in mice treated with Ly6G antibody, with female mice losing more bone than male mice (0.45 mm male, 0.52 mm female, *p* < 0.05, Figure 3E). Treatment with the Ly6G antibody decreased bone loss in male mice while increasing bone loss in female mice (Figure 3E). Anti-Ly6G treatment also eliminated dimorphism in volumetric analysis (Figure 3F). Histological examination of the ligature sites of antibody-depleted mice showed similar levels of tissue inflammation between male and female mice, including infiltration of inflammatory immune cells (Figure 3G) and inflammation in the mucosal tissue abutting the ligature (Figure 3H). These results were unaffected by delivery of the Ly6G antibody (Figures 3G,H).

**Figure 3:**
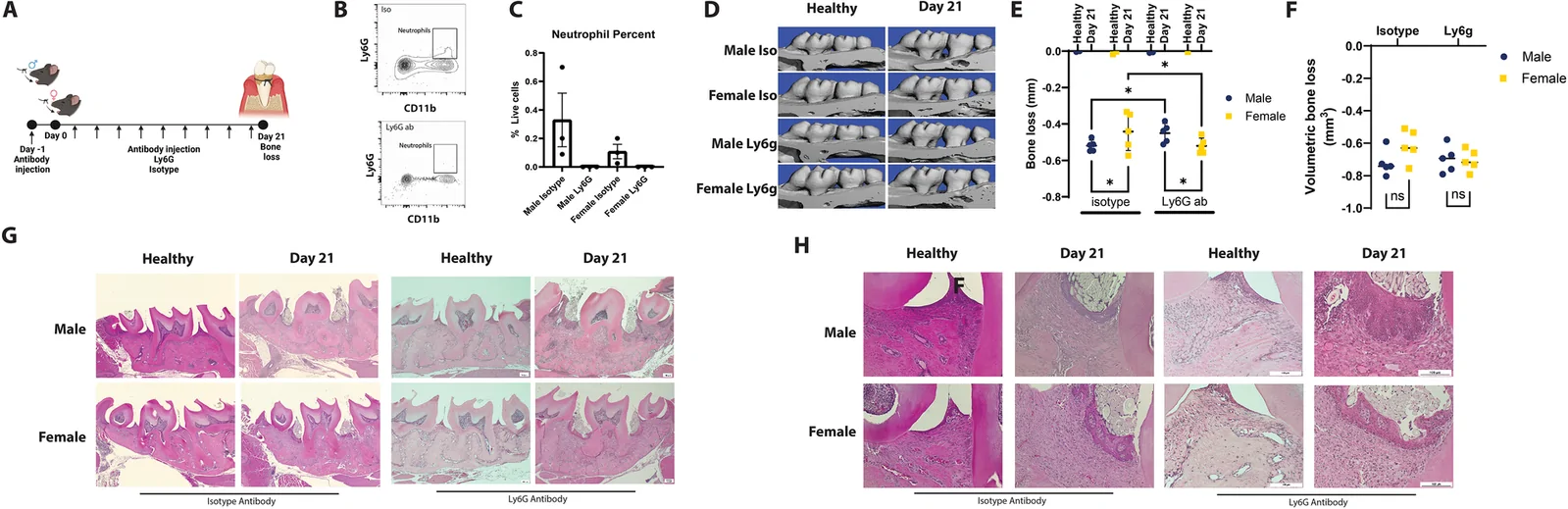
Neutrophil depletion differentially affects male and female mice. **(A)** Experimental plan for depletion of neutrophils during LIP. **(B)** Flow plots showing depletion of neutrophils in the blood of Ly6G antibody-treated mice but not isotype control-treated mice. **(C)** Quantification of neutrophil fraction in the blood during antibody treatment. **(D)** MicroCT images showing bone loss in isotype control mice at day 21. **(E)** Quantification of bone loss showing sexual dimorphism in isotype control-treated and Ly6G antibody-treated mice with LIP. **(F)** Volumetric quantification of maxillary bone loss. **(G)** Low-magnification histology image showing the ligature site in antibody-treated mice. Scale bar = 100 µM. H) High-magnification image of the ligature site in antibody-treated mice. Scale bar = 50 µM. *p < 0.05, two-way ANOVA.

### Human periodontitis patients have sexually dimorphic neutrophil signaling

Depletion of neutrophils differentially affected male and female mice. We therefore hypothesized that sexual dimorphism in neutrophil phenotype existed in periodontitis. We therefore examined sex differences in neutrophil phenotype in human periodontitis (Figure 4). To accomplish this, we aggregated publicly available single-cell RNA sequencing data (scRNAseq data) of individuals with and without periodontitis. Clustering this data using the Seurat algorithm (25) found 22 unique cell types within this data set (Figure 4A). Relative proportions of these unique cells were compared across all patient types (Figure 4). Granulocytes were identified as a cluster of cells with high expression of *CSF3R*, *FCG3RB, IL1B,* and *C5AR1* (Figure 4C). Next, we conducted cell-cell communication analysis using CellChat (26). Stromal elements in the oral mucosa (endothelial cells, fibroblasts, keratinocytes) were the primary sources of signaling molecules in both male and female periodontitis patients (Figure 4D). We next examined differences in signaling strength between pathways in male and female patients (Figure 4E). The SELL, CXCL, and COLLAGEN pathways were the most differential pathways based on inferred signaling strength. Plotting significantly contributing receptor-ligand interactions showed differences in incoming signals through *CD44* and *CXCL* receptors *CXCR1*, *CXCR2*, and *CXCR4* (Figure 4F). Outgoing differences were observed in *SELL*, *CXCL1*, and *CXCL8* (Figure 4G). CXCL1-ACKR1 interactions were specific to female neutrophils (outgoing from neutrophils to endothelial cells).

**Figure 4:**
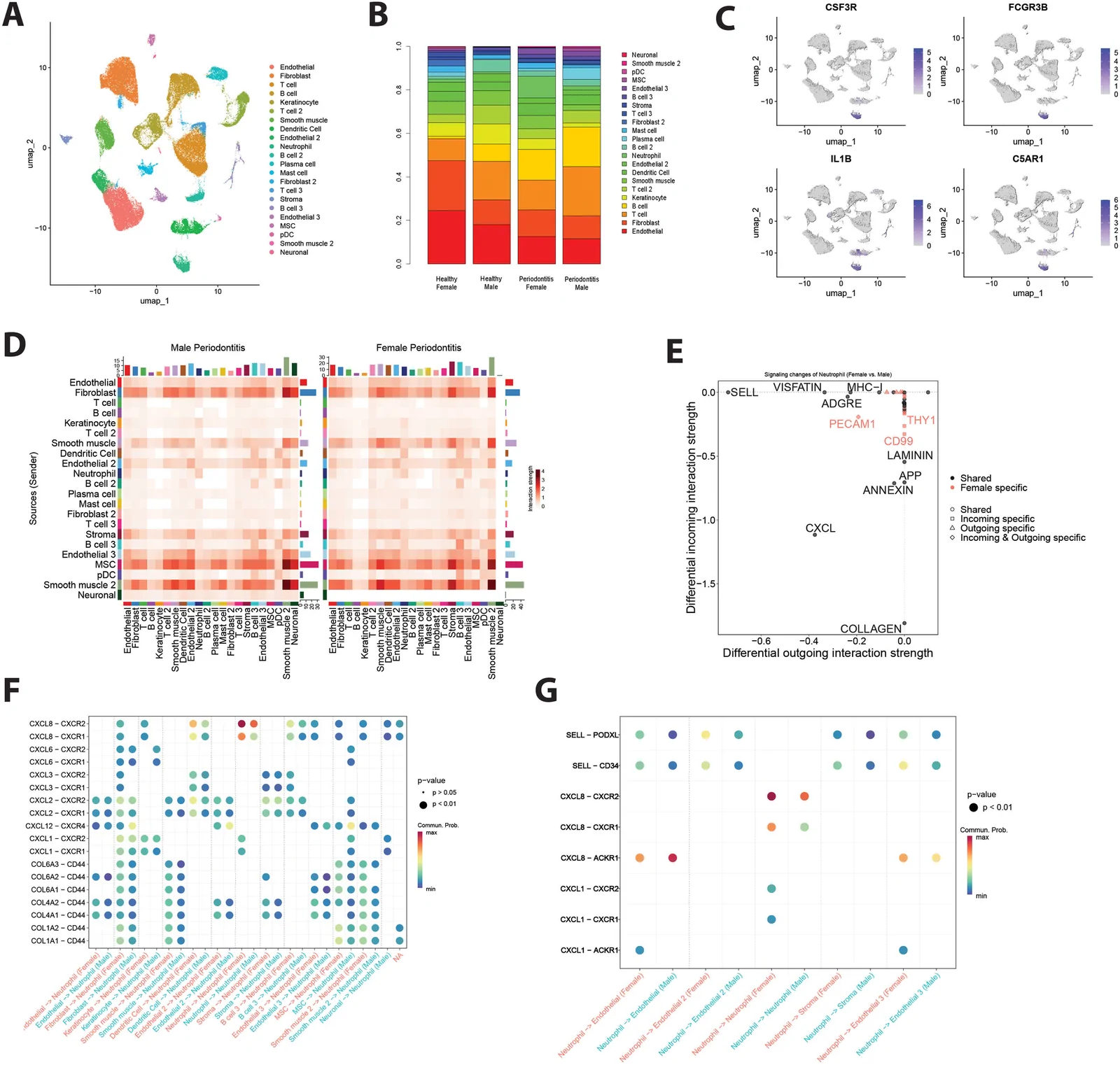
Neutrophil signaling in human periodontitis. **(A)** UMAP plot with labeled clusters from Seurat. **(B)** Relative proportions of different cells in healthy and periodontitis male and female patients. **(C)** Heatmap of granulocyte expression markers. **(D)** Signaling information heatmaps from CellChat for male and female patients with periodontitis. **(E)** Differential signaling networks based on incoming and outgoing interaction strength in male and female patients. **(F)** Specific receptor-ligand interactions with neutrophils as the receiver cluster. G) Specific receptor-ligand interactions with neutrophils as the sending cluster.

### Sex differences in neutrophil phenotypes during periodontitis

Reclustering neutrophils identified 10 unique cell clusters (Figure 5A). We compared gene expression across the different granulocyte clusters (Figure 5B, Supplementary Table S1). Comparing cluster distributions across conditions, we found Cluster 0 and Cluster 1 positively associated with periodontitis conditions, with granulocytes from female patients enriched in Cluster 0 and granulocytes from male patients enriched in Cluster 1 (Figure 5C). Cluster 0 was enriched for neutrophil markers *S100A9* and *SELL*. Cluster 1 was enriched for CXCR4 expression, with relatively low SELL expression. Gene set enrichment analysis confirmed upregulation of neutrophil-related gene sets in Cluster 0 relative to other granulocyte cells (Figure 5D). Cluster 1 was enriched for gene sets related to generalized cytokine signaling in the immune system (Figure 5E).

**Figure 5:**
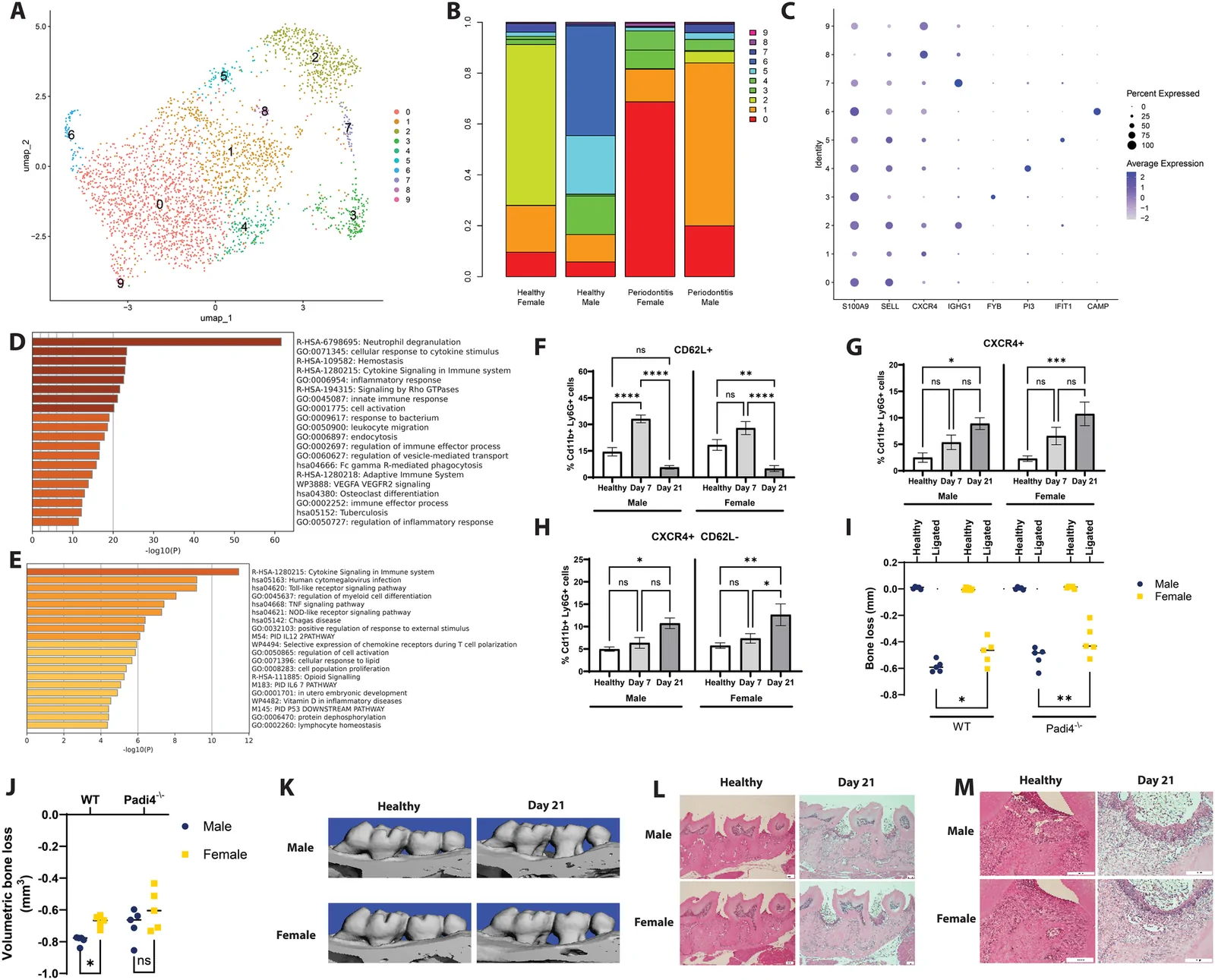
Neutrophil-specific phenotypes in periodontitis. **(A)** Reclustering of neutrophils using Seurat. **(B)** Relative expression of different neutrophil subtypes in healthy and periodontitis male and female patients. **(C)** Marker genes for different neutrophil subtypes in the oral mucosa of males and females with and without periodontitis. **(D)** Gene ontology of upregulated genes from Cluster 0. **(E)** Gene ontology of upregulated genes from Cluster 1. **(F)** Expression of CD62L in neutrophils from the maxillae of mice with different stages of LIP. **(G)** Expression of CXCR4 in neutrophils from the maxillae of mice with different stages of LIP. **(H)** Proportion of CD62L^−^ CXCR4^+^ neutrophils from the maxillae of mice with different stages of LIP. **(I)** Quantification of bone loss after 21 days of LIP in WT and Padi4 knockout mice. **(J)** Volumetric quantification of bone loss after 21 days of LIP in WT and Padi4 knockout mice. **(K)** MicroCT images of maxillae from Padi4 knockout mice with LIP. **(L)** Histology of entire ligature site. Scale bar = 100 µM. **(M)** Histology of ligature site. Scale bar = 50 µm. *p < 0.05, **p < 0.01, ***p < 0.001, ***p < 0.0001, two-way ANOVA with Tukey's multiple comparison correction.

Neutrophil extracellular trap (NET) production by infiltrating neutrophils has been previously shown to contribute to bone loss in periodontitis (29). Since neutrophils contributed significantly to sexual dimorphism in bone loss during periodontitis, we investigated the effect of neutrophil phenotype. We examined expression of CD62L (SELL) and CXCR4, two markers of NET-producing neutrophils (Figures 5F,G). CD62L expression peaked on day 7 in both male and female mice, with no significant difference between males and females (Figure 5F). CXCR4 expression peaked at day 21 with no difference between males and females (Figure 5G). We additionally characterized “aged” neutrophils, which have been described as CXCR4^+^, CD62L^−^ and are thought to produce more reactive oxygen species and NETs compared with CD62L^hi^ neutrophils (30, 31). Both male and female mice showed a statistically significant trend towards an increased CD62L^−^ CXCR4^+^ neutrophil fraction over time, with a peak at day 21 (Figure 5H). The relative proportion of these cells was the same between male and female mice throughout the experiment.

We next examined the effects of NET production on sexual dimorphism in periodontitis. This was accomplished by measuring bone loss during periodontitis in Padi4 knockout mice, which cannot produce NETs (32) (Supplementary S3). Male and female Padi4 knockout mice both lost statistically significant amounts of bone after ligature placement (Figures 5I,J); however, the amount of bone loss differed between males and females. Male Padi4 knockout mice had linear bone loss of 0.51 mm compared to 0.39 mm for female Padi4 knockout mice (*p* < 0.05). Similar sexual dimorphism was observed between WT and Padi4 knockout for both linear and volumetric bone loss, though volumetric was not significant (Figures 5I,J). Padi4 knockout mice showed less inflammation on histological characterization than WT mice (Figure 1G, Figures 5K,L). No differences were observed between male and female mice.

## Discussion

In this study, we demonstrate differential bone loss resulting from neutrophil-mediated inflammation in male and female mice. Sexual dimorphism of the skeleton is well documented (33). Mechanisms underlying these differences, particularly as they relate to alveolar bone, are not as thoroughly characterized. A variety of factors drive sexual dimorphism in the skeleton and bone homeostatic processes, including sex hormones, which play a fundamental role in determining both skeletal health as well as bone density-related pathologies (34–36). Sex hormones have also been shown to affect neutrophil function in a dimorphic manner. In uropathogenic *E. coli* urinary tract infections, increased testosterone levels induced prolonged polarization of macrophages toward the M2a phenotype, resulting in decreased bacterial clearance in the kidney and increased G-CSF and CXCL1 expression (37), which play crucial roles in neutrophil production and recruitment. Conversely, estrogen has been reported to inhibit neutrophil activation and chemotaxis (38, 39), specifically through E_2_ suppression of fMLP-induced ERK phosphorylation (38). Our data suggest that inherent differences in neutrophils, possibly driven by sex hormone signaling, though not manipulated here, may provide the basis for sexual dimorphism in the severity of periodontal disease.

Inflammation is a hallmark of periodontal disease (40) and discussions of sex differences in mechanisms of disease progression must include innate immune cells. Toll-like receptor 7 (TLR7) is encoded on the X chromosome and may escape X-inactivation in certain cell types, leading to higher levels of TLR7 in female cells relative to male counterparts (41, 42). Toward this end, TLR signaling pathways yield sexually dimorphic responses, including higher cytokine production in females for myeloid primary response gene 88 (MYD88), retinoic acid-inducible gene-I (RIGI), interferons, Janus kinase 2(JAK2), signal transducer and activator of transcription 3 (STAT3), NF-*κ*B, interferon gamma (IFN-*γ*), and tumor necrosis factor alpha (TNF) (43). Compared with females, peritoneal macrophages from males express higher levels of TLR4, a receptor for bacterial wall lipopolysaccharides (LPS) that induces large-scale cellular inflammatory responses in the oral mucosa, and show more robust levels of CXCL10 (44). Notably, female macrophages have enhanced phagocytosis and antigen-presentation capacity to T-lymphocytes, initiating the adaptive immune response (44). Sexual dimorphism during immune activation has been well established, although reports defining the mechanistic role these differences play in the progression of periodontal disease are limited. A model of periodontitis induced by *A. actinomycetemcomitans*-derived LPS enhanced RANKL-induced osteoclastogenesis in male mice relative to female mice (45, 46). The genes *Nfatc1* and *Tm7sf4* (encoding dendritic cell-specific transmembrane protein or DCSTAMP) were also more highly expressed in male osteoclasts in this model, suggesting an innate immune-driven mechanism for these differences (45, 46). There are sex-based differences in innate, humoral, and cellular immune recruitment and function in humans, although the exact mechanisms that drive this notable contrast remain insufficiently characterized (47). In our studies, innate immune activation appeared to contribute to sex-specific differences in hematopoiesis during chronic periodontitis.

Neutrophils are the most abundant innate immune cell in oral mucosal tissues and barrier sites such as the gingiva (23). They are key mediators of health and disease in the periodontal space within the oral cavity. In their absence or when they are functionally defective, as is the case in certain congenital disorders, severe forms of periodontitis develop at early ages (48). However, the presence of supernumerary or hyper-responsive neutrophils leads to imbalanced host-microbe interactions that culminate in dysbiosis and inflammatory tissue breakdown (49). Healthy young adult males displayed significant enhancement in immature neutrophil gene signatures compared to age-matched females, resulting in 1) a less activated male neutrophil phenotype based on cell surface markers, 2) a hampered response to cytokine stimulation, and 3) decreased ability to form neutrophil extracellular traps (NETs) following stimulus (50). Conversely, healthy young adult females have an activated/mature neutrophil profile characterized by enhanced type I IFN pathway activity and enhanced proinflammatory responses (51). We demonstrated that experimental periodontitis increased neutrophil infiltration in males, and hypothesized that neutrophil depletion would eliminate the observed differences in bone loss. While neutrophil depletion significantly reduced bone loss in males compared, female mice lost more bone than male mice (Figure 3). Ly6G inhibition *in vivo* has been shown to stimulate bone resorption in mice by depleting circulating mature neutrophils and reducing the number of proliferating preneutrophils, which likely inhibit osteoclastogenesis (52). In previous work, we demonstrated that inhibition of NET formation using Padi4 knockout mice greatly reduced cortical and trabecular bone of the tibia in male mice, but not females (53). This suggests neutrophil function, not number, may be responsible for increased bone loss in males. However, impaired NET function did not significantly affect differences in bone loss between male and female mice, indicating that other functions, such as reactive oxygen species production and other proinflammatory cytokines, likely drive this phenotype.

The studies presented here used a single animal model and do not directly investigate hormone manipulation in the observed bone loss phenotype. We also recognize that factors such as the oral microbiome, while mentioned in the context of hyper-responsive neutrophils, were not investigated in this study and therefore cannot be excluded. A recent meta-analysis evaluating the sexually dimorphic oral microbiome found that females with periodontitis had increased abundance of several periodontal pathogens in the subgingival plaque compared to men (54). This could explain why neutrophil depletion resulted in increased linear bone loss in female mice, but more thorough investigation is required to evaluate the potential contribution. Contributions from factors such as biting forces or trauma from occlusion were also not evaluated. Primary trauma from occlusion may occur from excessive biting forces on a tooth with healthy or normal bone support (55). Secondary trauma includes injury by either normal or excessive bite forces on a tooth with reduced periodontium (55), which may be more relevant as periodontal disease and tissue destruction are occurring.

## Conclusion

Sexual dimorphism can be observed in the clinical phenotype of experimental periodontitis. Differences in bone loss are driven, in part, by differences in neutrophil recruitment and phenotype at the site of inflammation. Future therapies could target these differences to personalize periodontal treatment.

## Data Availability

The raw data supporting the conclusions of this article will be made available by the authors, without undue reservation.
